# Development of the interpretable typing prediction model for osteosarcoma and chondrosarcoma based on machine learning and radiomics: a multicenter retrospective study

**DOI:** 10.3389/fmed.2024.1497309

**Published:** 2024-11-20

**Authors:** Qing-Yuan Long, Feng-Yan Wang, Yue Hu, Bo Gao, Chuan Zhang, Bo-Heng Ban, Xiao-Bin Tian

**Affiliations:** ^1^The Second Affiliated Hospital of Guizhou Medical University, Kaili, China; ^2^School of Clinical Medicine, Guizhou Medical University, Guiyang, China; ^3^Guang’anmen Hospital, China Academy of Chinese Medical Sciences, Beijing, China; ^4^Qiannan State Hospital of Traditional Chinese Medicine, Duyun, China

**Keywords:** osteosarcoma, chondrosarcoma, machine learning, typing prediction, interpretability

## Abstract

**Background:**

Osteosarcoma and chondrosarcoma are common malignant bone tumors, and accurate differentiation between these two tumors is crucial for treatment strategies and prognosis assessment. However, traditional radiological methods face diagnostic challenges due to the similarity in imaging between the two.

**Methods:**

Clinical CT images and pathological data of 76 patients confirmed by pathology from January 2018 to January 2024 were retrospectively collected from Guizhou Medical University Affiliated Hospital and Guizhou Medical University Second Affiliated Hospital. A total of 788 radiomic features, including shape, texture, and first-order statistics, were extracted in this study. Six machine learning models, including Random Forest (RF), Extra Trees (ET), AdaBoost, Gradient Boosting Tree (GB), Linear Discriminant Analysis (LDA), and XGBoost (XGB), were trained and validated. Additionally, the importance of features and the interpretability of the models were evaluated through SHAP value analysis.

**Results:**

The RF model performed best in distinguishing between these two tumor types, with an AUC value close to perfect at 1.00. The ET and AdaBoost models also demonstrated high performance, with AUC values of 0.98 and 0.93, respectively. SHAP value analysis revealed significant influences of wavelet-transformed GLCM and First Order features on model predictions, further enhancing diagnostic interpretability.

**Conclusion:**

This study confirms the effectiveness of combining machine learning with radiomic features in improving the accuracy and interpretability of osteosarcoma and chondrosarcoma diagnosis. The excellent performance of the RF model is particularly suitable for complex imaging data processing, providing valuable insights for the future.

## 1 Introduction

Osteosarcoma, a malignant tumor originating from mesenchymal stem cells, has an annual incidence rate ranging from 1 to 4 cases per million people and can occur at any age, with a higher prevalence in children and adolescents (1). These tumors primarily affect long bones such as the distal femur, proximal tibia, and proximal humerus (2). Although the exact etiology of osteosarcoma remains unclear, factors such as abnormal bone tissue proliferation and exposure to radiation may be associated with its development (3). Treatment typically includes neoadjuvant chemotherapy, surgery, and high-dose chemotherapy (4). Despite 5-year survival rates ranging from 37.5 to 77.6% for patients with localized osteosarcoma after systemic therapy (5–9), approximately 20–30% of patients experience metastasis, primarily to the lungs and bones. For metastatic or recurrent osteosarcoma, adjuvant chemotherapy has not significantly improved survival rates (10). In contrast, treatment outcomes for chondrosarcoma, another common malignant bone tumor, are generally more favorable. Both osteosarcoma and chondrosarcoma typically develop within bone tissue, and their lesions often share similarities in location, morphology, and texture during bone formation. This makes accurate diagnosis and differentiation particularly challenging. Precise diagnosis is critical, as misdiagnosis can result in patients missing the optimal treatment window or undergoing unnecessary treatments.

Although percutaneous biopsy remains the gold standard for osteosarcoma diagnosis, it is an invasive procedure that may lead to complications such as bleeding, infection, or tumor dissemination (11, 12). In recent years, with the rapid development of medical imaging technology, computed tomography (CT) has become an important tool for diagnosing osteosarcoma and chondrosarcoma. CT technology, with its high-resolution imaging capability, can provide detailed information about tumor size, morphology, location, and whether it invades surrounding tissues, which is crucial for early detection and staging of the disease. Furthermore, CT scans can reveal subtle changes in bone structure and soft tissue conditions, which are essential for accurately assessing tumor invasiveness and potential metastasis. However, the high variability of these tumor types and their similarity to other lesions make traditional manual imaging diagnostics not only time-consuming but also heavily reliant on the experience and expertise of radiologists. This adds to the complexity of diagnosis and can sometimes result in misdiagnosis or missed cases. Thus, there is an urgent need for the development of automated, accurate, and efficient imaging-based diagnostic tools.

The rise of machine learning techniques has transformed the processing and analysis of medical images, making precise prediction of pathological types possible. Particularly in the field of radiomics, this technology, through high-throughput data feature extraction algorithms, can convert complex medical images into high-dimensional, usable quantitative image features. These features are then used to build machine learning models that can not only differentiate between benign and malignant tumors but also predict the prognosis of tumor patients (13–15). For example, by analyzing features such as texture, shape, and density in CT images, machine learning models can accurately identify osteosarcoma and chondrosarcoma and even predict their developmental trends and potential treatment responses. This enables doctors to make more accurate clinical decisions in a shorter amount of time, which is crucial for enhancing patient treatment outcomes.

**Figure 1:**
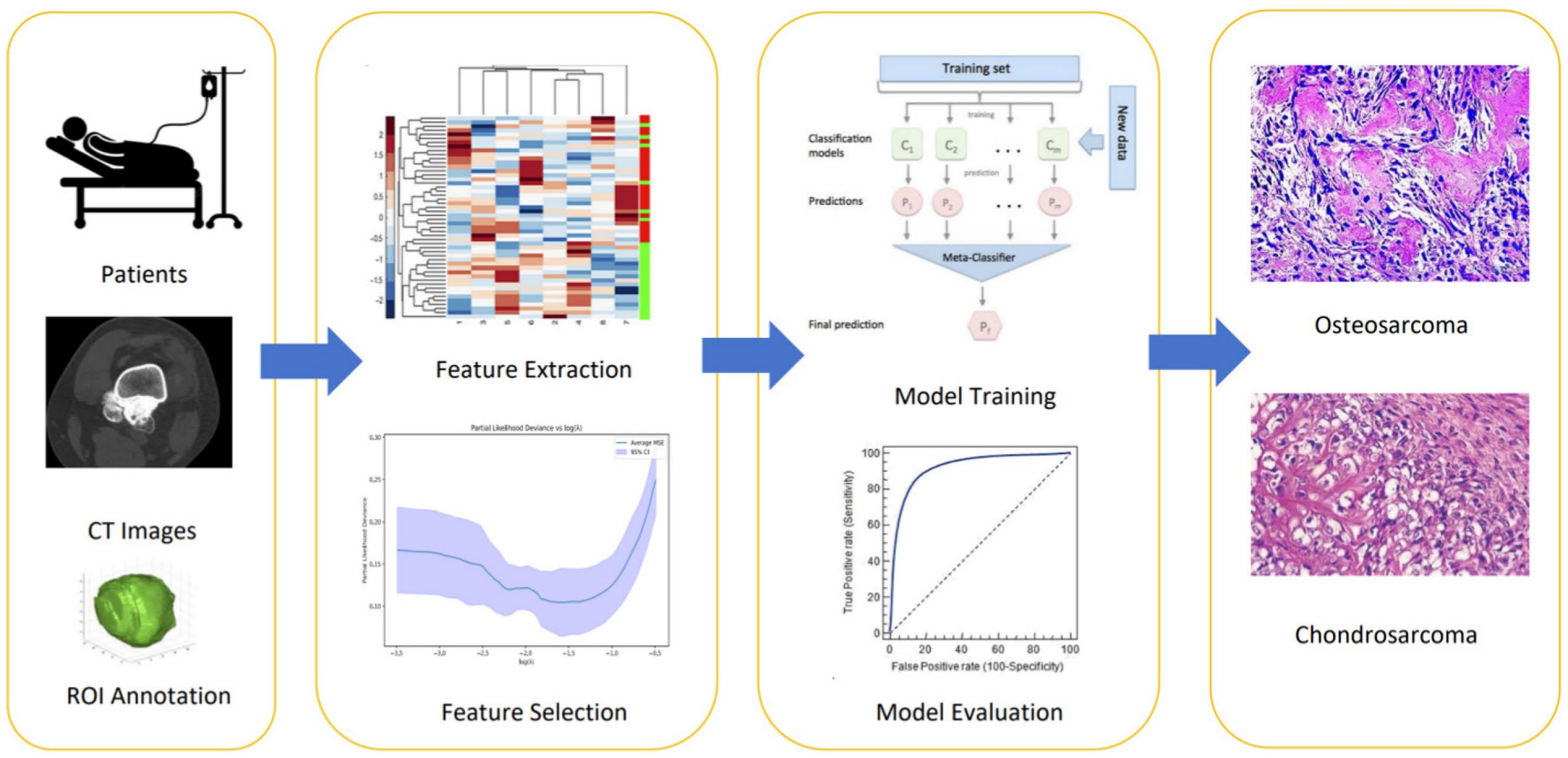
The workflow of this study includes: (1) collecting CT images and performing manual ROI annotation to define tumor regions; (2) extracting radiomic features such as shape, texture, and first-order statistics; (3) using LASSO regression for feature selection; (4) training machine learning models on selected features to differentiate osteosarcoma from chondrosarcoma; and (5) evaluating model performance with metrics like AUC to select the best model for clinical interpretation.

This study, based on retrospective CT data from two medical centers, aims to develop an innovative machine learning and radiomics-based model for distinguishing between osteosarcoma and chondrosarcoma. By integrating tumor imaging data with pathological diagnostic results, this study applied six advanced machine learning algorithms to process and analyze data, thereby extracting imaging features that can clearly differentiate between the two tumors. The collected CT images were preprocessed, including standardization and enhancement, to improve the quality and efficiency of model training. Subsequently, high-throughput data feature extraction techniques were used to extract multidimensional quantitative features from CT images, such as texture, shape, and density. After training and validation, this study ultimately developed a machine learning model capable of accurately distinguishing between osteosarcoma and chondrosarcoma, offering promise as a novel clinical auxiliary diagnostic tool. The workflow of this study is shown in Figure 1.

## 2 Methods

### 2.1 Patient enrollment

This study adhered to the Helsinki Declaration and obtained approval from the Ethics Committees of Guizhou Medical University Affiliated Hospital (Approval No.:) and Guizhou Medical University Second Affiliated Hospital (Approval No.:). Retrospectively, CT images of patients diagnosed with osteosarcoma by osteosarcoma resection surgery were collected from the two centers, Guizhou Medical University Affiliated Hospital and Guizhou Medical University Second Affiliated Hospital, from January 2018 to January 2023. A total of 76 patients were included, comprising 36 males and 40 females. According to pathological classification criteria, they were divided into the chondrosarcoma group (*n* = 31) and the osteosarcoma group (*n* = 45). Inclusion criteria were as follows: (1) Patients diagnosed with sarcoma and undergoing resection surgery; (2) Patients undergoing preoperative CT scanning; (3) Patients with complete clinical data. Exclusion criteria were as follows: (1) Obvious artifacts on CT images; (2) Patients with tumors in addition to sarcoma; (3) Patients who had not received treatment before CT scanning.

### 2.2 Imaging examination

In this study, CT images of all patients were acquired from the same CT scanner, model GE Revolution CT 256. To ensure image quality and consistency, all scans were performed under uniform technical conditions. Patients were positioned in a supine position during CT scanning to minimize motion artifacts and ensure image clarity and accuracy.

The specific scan range covered the joints where the sarcoma was located and its adjacent joints above and below to comprehensively evaluate the local extension of the tumor and possible joint involvement. The specific parameters for the scans were set as follows: tube voltage was set to 120 kV to optimize image contrast and reduce radiation dose; tube current was adjusted between 250 and 400 mA based on patient size to ensure image quality; slice thickness and slice spacing were both set to 5 mm to improve spatial resolution of the images, making subtle bone destruction and soft tissue structure changes more clearly visible; the image matrix was set to 512 × 512 to ensure sufficient image detail; detector collimation was 64 mm × 0.625 mm, ensuring efficient data acquisition and shorter scan times.

The CT scanning procedure began with obtaining plain images of the patient’s abdomen to preliminarily evaluate the anatomical structures in the abdominal region and exclude other potential complications. Subsequently, detailed scans of the sarcoma area were performed to accurately assess the size, morphology, and relationship with surrounding structures of the tumor.

### 2.3 Data preprocessing

To ensure that machine learning models could effectively learn and accurately predict osteosarcoma and chondrosarcoma, strict preprocessing of the collected CT image data was conducted. The main objectives of data preprocessing were to improve data quality, reduce the influence of noise, and create a uniform format suitable for subsequent analysis and model training.

All image data underwent initial cleaning to remove any artifacts or images from non-tumor areas generated during scanning. Specificity, we performed preliminary cleaning on all CT images to remove artifacts or images of non-tumor areas caused by the scanning process. In order to ensure data quality, we removed any images with motion artifacts or unclear annotations to ensure that all images used for subsequent analysis are of high quality and accurately labeled.

Subsequently, image standardization was performed to eliminate the influence of different scanning parameters on image intensity. We standardized the grayscale values of all CT images to eliminate the effects of different scanning parameters and equipment on image intensity. This standardization process is completed by scaling the grayscale values of the image to a range of 0 to 255. Specifically, we use the minimum and maximum grayscale values of each image for linear scaling to ensure that the grayscale values between different images are in the same range, which is convenient for subsequent feature extraction and model training. This step was crucial for the model to fairly compare and process images from different scans.

To further enhance the visibility of tumor features in the images, various image enhancement techniques were applied. This included contrast enhancement, sharpening, and noise reduction algorithms, which helped highlight details in the tumor area, facilitating the model to more accurately learn and identify tumor-related image features.

To ensure accurate extraction of radiomic features and accuracy of subsequent analysis, all CT images involved in this study underwent manual Region of Interest (ROI) annotation. ROI annotation was performed using 3D Slicer software. During the ROI annotation process, two radiologists with over 10 years of experience jointly delineated each image to ensure the accuracy and consistency of the annotations. The main objective of annotation was to precisely define the regions showing tumor features, including the main body of the tumor and possible invasion margins. Subsequently, to further improve the accuracy of the annotations, all annotated images underwent review and adjustment by another physician before final analysis. This process ensured the representativeness and relevance of the extracted features.

### 2.4 Radiomic feature extraction and selection

#### 2.4.1 Feature extraction

In this study, to extract reliable radiomic features from CT images of osteosarcoma and chondrosarcoma, we utilized the pyradiomics library, which is a widely used feature extraction tool for medical imaging data. We used pyradiomics to extract features from each preprocessed CT image, covering various aspects such as shape, texture, and signal intensity. Specifically, these features included first-order statistics, shape-based features, Gray Level Co-occurrence Matrix (GLCM), Gray Level Run Length Matrix (GLRLM), Gray Level Dependence Matrix (GLDM), and Gray Level Size Zone Matrix (GLSZM) parameters. Pyradiomics applies mathematical operations to generate different feature sets. For example, first-order statistics are computed directly from the voxel intensity values within the ROI, providing basic information about the distribution of intensities (e.g., mean, standard deviation, and entropy). Shape features are extracted by analyzing the three-dimensional structure of the tumor, focusing on parameters such as volume, surface area, and sphericity. To capture more complex patterns, pyradiomics then generates texture features using advanced matrices like the Gray Level Co-occurrence Matrix (GLCM) and Gray Level Run Length Matrix (GLRLM), which quantify spatial relationships between pixel intensities, offering deeper insight into the tumor’s internal structure.

#### 2.4.2 Feature selection

Feature selection was conducted in several steps. Firstly, the stability of features was assessed using the Intraclass Correlation Coefficient (ICC). Only features with an ICC value greater than 0.75 were considered to have sufficient test–retest stability and were suitable for subsequent analysis. Next, all high-ICC features were standardized using Z-score normalization to eliminate biases due to scale and magnitude. Following standardization, features significantly correlated with disease status were selected using t-tests, where those features showing a *p*-value less than 0.05 were considered significantly different between osteosarcoma and chondrosarcoma samples. Finally, Lasso regression model was applied for further feature selection. Lasso regression adds an L1 regularization term to shrink some coefficients to zero, effectively selecting the most important features and reducing model complexity. This helps prevent overfitting and improves prediction accuracy. We used cross-validation to optimize the regularization parameter (λ), which determines the strength of the penalty. By evaluating different λ values, we selected the one that minimized prediction error while retaining the most relevant features, enhancing both model performance and interpretability. This set of features was used for subsequent machine learning algorithm training to construct an efficient and accurate osteosarcoma prediction model.

### 2.5 Construction of machine learning models

To accurately differentiate between osteosarcoma and chondrosarcoma, this study employed six mainstream machine learning models, namely XGBoost (XGB), Random Forest (RF), Extra Trees (ET), Gradient Boosting (GB), AdaBoost, and Linear Discriminant Analysis (LDA). Each model was optimized and validated under a series of predefined parameters.

#### 2.5.1 XGBoost

XGB is an optimized distributed gradient boosting library based on gradient boosting algorithm. In this study, key parameters for XGB were set as follows: “max_depth = 5”, limiting the maximum depth of trees to control overfitting; “learning_rate = 0.1,” controlling the contribution of each tree; “n_estimators = 100,” defining the total number of trees to be constructed; “subsample = 0.8”, used for random sampling of training instances; “colsample_bytree = 0.8,” used for random sampling of features.

#### 2.5.2 Random forest

RF is an ensemble learning method with good handling capability for high-dimensional data. Parameters for the RF model include: “n_estimators = 100,” indicating the number of trees in the forest; “max_features = ‘sqrt’,” considering the number of features to look for when finding the best split; “min_samples_split = 2,” requiring at least two samples in a node for splitting; “min_samples_leaf = 1,” requiring at least one sample in a leaf node.

#### 2.5.3 Extra trees

The ET classifier is similar to RF but more random in its splitting strategy at each node. Model parameters for ET are set as: “n_estimators = 100,” “max_features = ‘auto’,” automatically selecting the number of features; “min_samples_split = 2,” and “min_samples_leaf = 1.”

#### 2.5.4 Gradient boosting

GB is a method of precisely adjusting the model by progressively adding models to reduce bias. Parameters for the GB model are set as: “n_estimators = 100,” “learning_rate = 0.1,” “max_depth = 3,” controlling the depth of each tree; “subsample = 0.8,” and “max_features = ‘sqrt’.”

#### 2.5.5 AdaBoost

AdaBoost is a technique for boosting models by adjusting the weights of misclassified instances by the previous model. Parameters for AdaBoost are set as: “n_estimators = 50,” “learning_rate = 1.0.”

#### 2.5.6 Linear discriminant analysis

LDA is a technique for pattern classification and is well-suited for handling highly collinear data. Due to its fewer parameters, LDA was used as a baseline model.

### 2.6 Statistics and analysis

In this study, we evaluated and compared different machine learning models with the aim of accurately distinguishing between osteosarcoma and chondrosarcoma. The experimental data were divided into training and testing sets in an 8:2 ratio, where the training set was used for model construction and parameter optimization, and the testing set was used for final model performance evaluation. All experiments were conducted on a high-performance computer equipped with Windows 10 operating system, Intel Core i9 processor, and NVIDIA GeForce RTX 3080 graphics card. Additionally, this study was developed using Python 3.7 programming language, primarily relying on data science and machine learning libraries such as Scikit-learn, XGBoost, Pandas, and Numpy. For statistical analysis, this study utilized evaluation metrics including accuracy (Acc), recall (Recall), precision (Prec), F1 score, and AUC value, and detailed analysis of model performance was conducted through confusion matrices.

## 3 Results

### 3.1 Baseline characteristics of patients

The clinical baseline characteristics of patients from the Guizhou Medical University Affiliated Hospital and the Guizhou Medical University Second Affiliated Hospital are summarized in Table 1. At the Guizhou Medical University Affiliated Hospital, the average age of osteosarcoma patients (*N* = 39) was 22.87 ± 14.663 years, which was significantly younger compared to chondrosarcoma patients (*N* = 27), whose average age was 47.67 ± 13.890 years (*p*-value < 0.01). At the Guizhou Medical University Second Affiliated Hospital, the average age of osteosarcoma patients (*N* = 6) was 23.83 ± 11.822 years, while the average age of chondrosarcoma patients (*N* = 4) was 42.25 ± 13.841 years (*p*-value = 0.068), showing a similar trend but without reaching statistical significance. Regarding gender distribution, at the Guizhou Medical University Affiliated Hospital, 48.7% of the osteosarcoma patients and 44.4% of the chondrosarcoma patients were male (*p*-value = 0.732). At the Guizhou Medical University Second Affiliated Hospital, 50% of the osteosarcoma patients and 50% of the chondrosarcoma patients were male (*p*-value = 1.000), indicating no significant difference in gender distribution between the two groups at both hospitals.

**Table 1:** Clinical baseline characteristics statistics.

	Guizhou Medical University Affiliated Hospital (*N* = 66)			Guizhou Medical University Second Affiliated Hospital (*N* = 10)		
	Osteosarcoma (*N* = 39)	Chondrosarcoma (*N* = 27)	*p*-value	Osteosarcoma (*N* = 6)	Chondrosarcoma (*N* = 4)	*p*-value
Age, years	22.87 ± 14.663	47.67 ± 13.890	<0.01	23.83 ± 11.822	42.25 ± 13.841	0.068
Men, *n* (%)	19 (48.7)	12 (44.4)	0.732	3 (50)	2 (50)	1.000

### 3.2 Feature extraction and selection results of radiomics

In this study, a total of 788 radiomic features were extracted, including 100 features obtained from original transformations and 688 features extracted through wavelet transformation. In the original transformations, extracted contents comprised 14 shape features, 18 first-order statistical features, 22 gray-level co-occurrence matrix (GLCM) features, 16 gray-level run-length matrix (GLRLM) features, 16 gray-level size zone matrix (GLSZM) features, and 14 gray-level dependence matrix (GLDM) features. In the wavelet transformation, 144 first-order statistical features, 176 GLCM features, 128 GLRLM features, 128 GLSZM features, and 112 GLDM features were extracted through different wavelet decompositions (Wavelet-LLH, Wavelet-LHL, Wavelet-LHH, Wavelet-HLL, Wavelet-HLH, Wavelet-HHL, Wavelet-HHH, and Wavelet-LLL).

**Figure 2:**
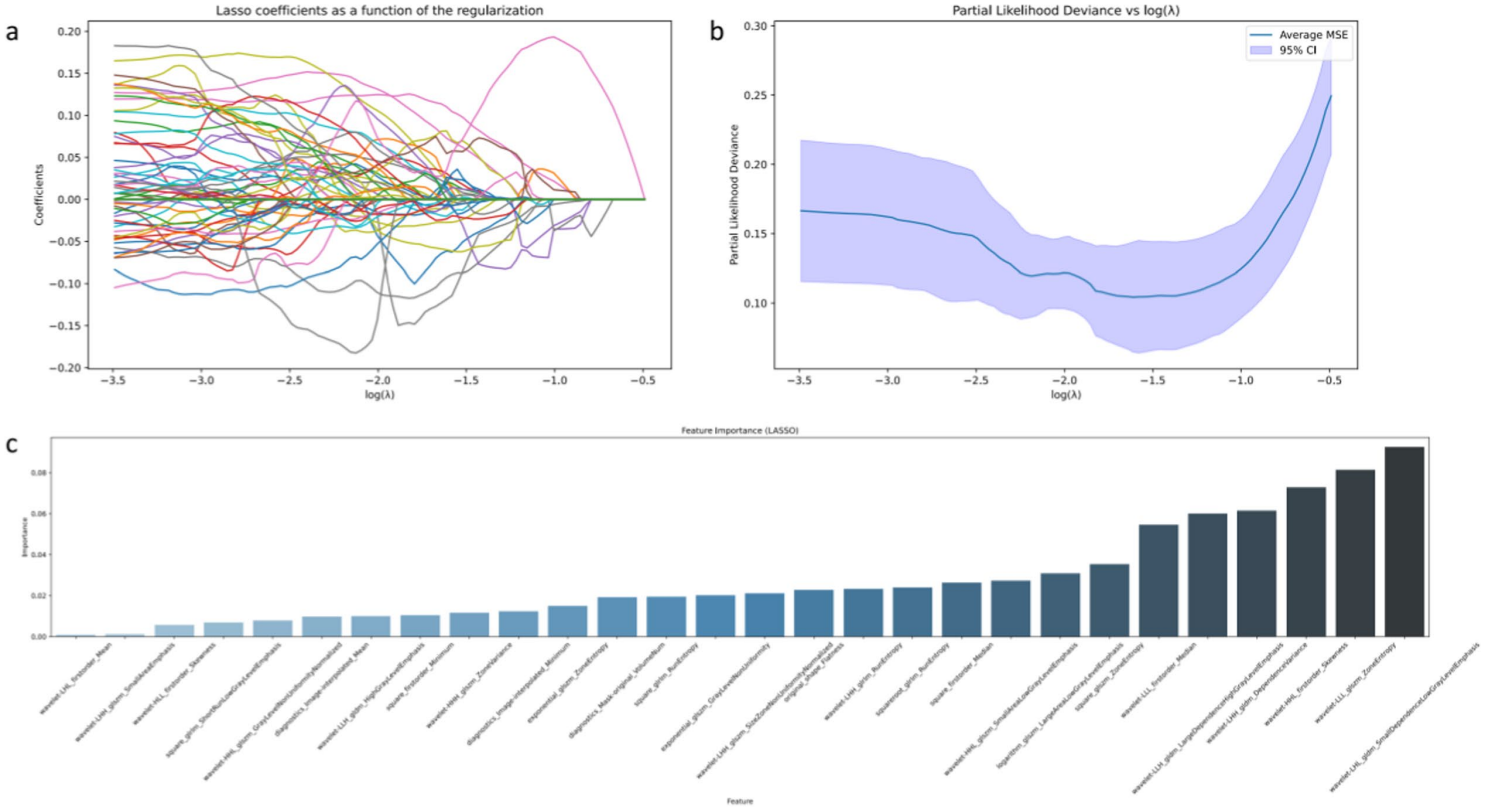
Visual display of radiomic features filtered by LASSO algorithm. Among them (a) represents the changing trend of the Lasso coefficient of each feature as the regularization parameter *λ* increases; (b) the model deviation under different regularization parameters λ; (c) represents the importance ranking of features filtered by LASSO.

The results of feature selection using Lasso regression indicated that some features significantly contributed to the predictive performance of the model. Lasso regression effectively selected features with significant impact on classification by introducing L1 penalty, achieving sparse selection of features. From Figures 2a–c, detailed analysis and interpretation of the results of Lasso feature selection can be observed. Figure 2a illustrates how the Lasso coefficients of each feature change with increasing regularization parameter *λ*. It can be observed that at smaller λ values, most feature coefficients are relatively large, gradually decreasing toward zero as λ increases. This indicates that under lower penalty strength, most features contribute to the model to some extent, whereas under strong regularization, only a few key features are retained. Figure 2b shows the relationship between bias and regularization strength. It can be seen that as λ decreases, bias decreases, suggesting better data fitting performance at smaller λ values (i.e., lower regularization strength). In Figure 2c, an uneven distribution of feature importance is observed, particularly with wavelet transformation features occupying a significant position in feature importance. For instance, “wavelet.LLH_glcm_Idn,” “wavelet.HLH_firstorder_Skewness,” and “wavelet.LHH_glcm_Idm” exhibit high importance, indicating their high predictive ability in distinguishing between osteosarcoma and chondrosarcoma.

### 3.3 Results of machine learning-based predictive models

**Figure 3:**
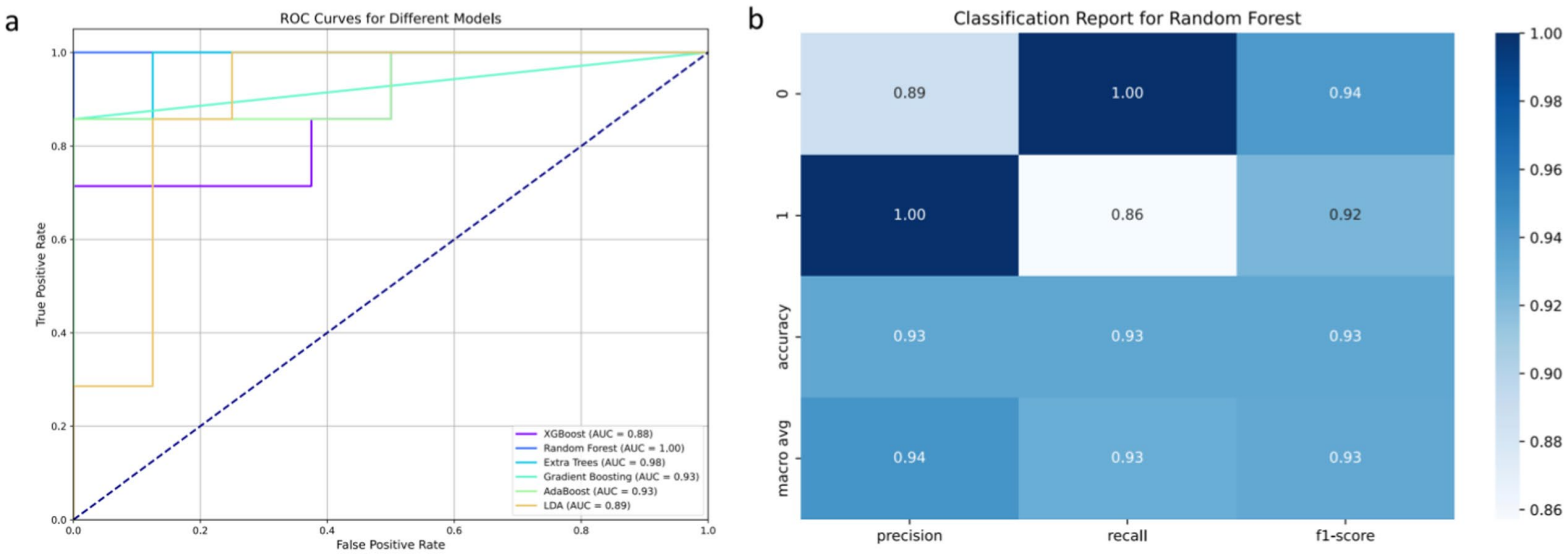
Results of osteosarcoma prediction model based on machine learning model. Among them (a) represents the comparison of ROC curves of six machine learning algorithms; (b) represents the matrix display of various evaluation indicators of the selected RF algorithm.

The results of the osteosarcoma prediction model are illustrated in Figure 3. Figure 3a analysis demonstrates that various machine learning models exhibit varying degrees of diagnostic efficacy in distinguishing between osteosarcoma and chondrosarcoma. The RF model demonstrates the highest discriminatory ability, with an AUC value reaching a perfect 1.00, indicating its ability to accurately differentiate between case types under all test conditions. The ET model also demonstrates near-perfect performance, with an AUC of 0.98, suggesting its effective discrimination between the two tumors in most scenarios. AdaBoost and GB models also exhibit strong predictive capabilities, with AUC values of 0.93, indicating high reliability and diagnostic accuracy. Relatively, although slightly weaker, the LDA and XGBoost models still demonstrate good diagnostic capabilities, with AUC values of 0.89 and 0.88, respectively, providing valuable classification decision support within an acceptable range.

The classification report in Figure 3b further reveals the advantages of the RF model in specific classification tasks. In distinguishing between the two classes of cases, the model achieves precision and recall rates of 0.93 each, with an F1 score of 0.93, demonstrating high consistency and reliability in handling such medical imaging data. This high level of performance is attributed to the RF model’s ability to balance well between processing high-dimensional data and achieving good performance across different categories.

### 3.4 Interpretability analysis of radiomic features

**Figure 4:**
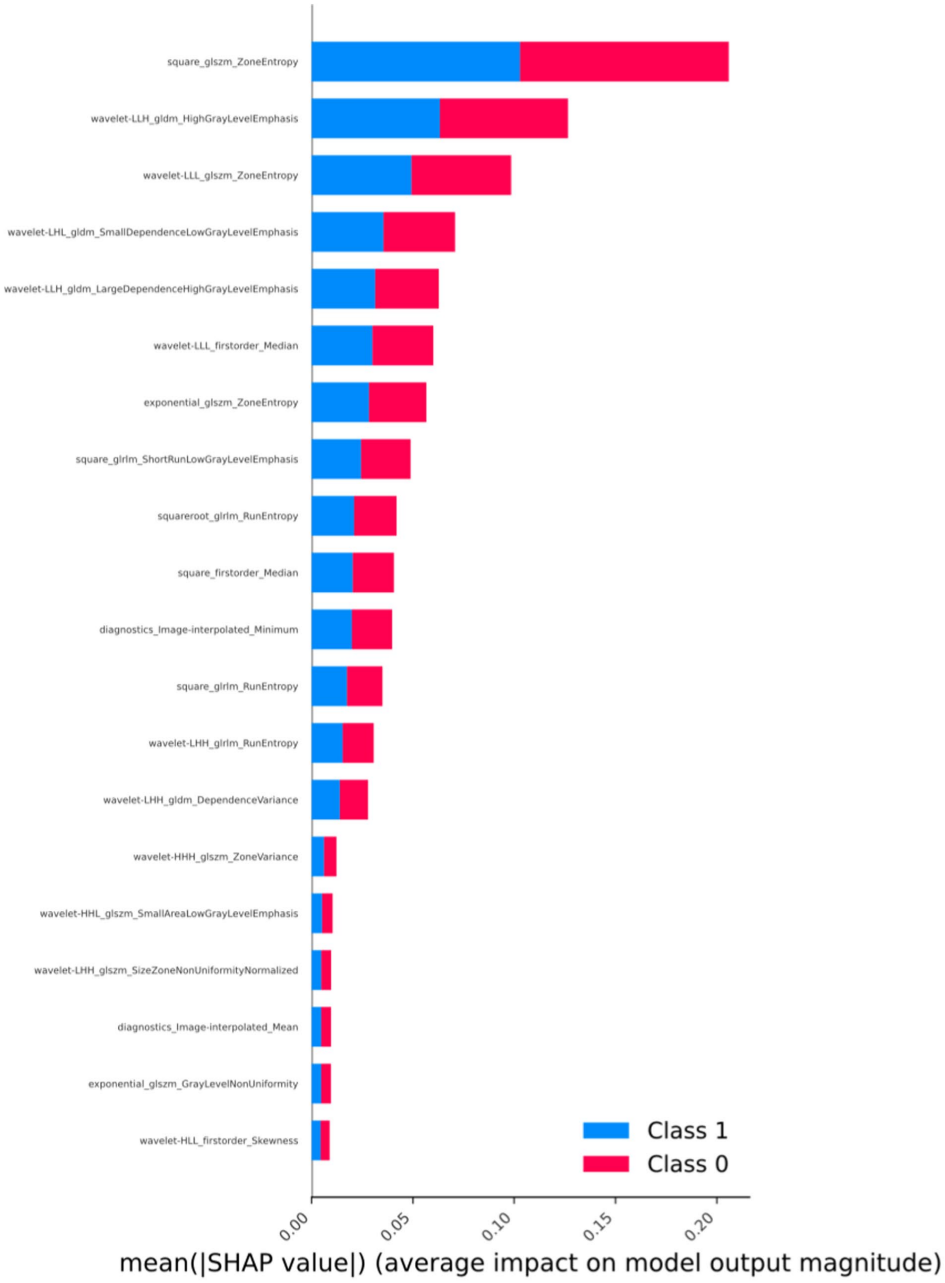
Arrangement of feature importance that affects the model (SHAP analysis, taking the best-performing model as an example).

The importance ranking of radiomic features is depicted in Figure 4. Through a detailed analysis of SHAP values, we can establish connections between abstract radiomic features and specific characteristics of osteosarcoma, providing more specific insights for clinical diagnosis. In the SHAP value analysis, we observe that certain wavelet transformation features, such as “wavelet-LHL_gldm_DependenceLowGrayLevelEmphasis” and “wavelet-HLH_glcm_JointAverage,” significantly influence the model’s predictive output. “DependenceLowGrayLevelEmphasis” describes the pixel dependency of low gray-level regions in the image. In the context of osteosarcoma imaging, this may indicate the boundary between low-density areas of tumor tissue and surrounding tissues, which could represent necrotic areas of the tumor or tissue with less vascular support, common features of malignant tumors. The strong performance of this feature may aid in distinguishing highly heterogeneous malignant tumors from more uniform benign or less malignant tumors.

On the other hand, “JointAverage,” as a first-order statistical feature, provides information about the average pixel intensity of the entire image region or tumor area. In the context of tumors, this may reflect the general characteristics of tumor texture. For instance, higher “JointAverage” values may correspond to denser tumor tissue, which is more common in certain types of high-density tumors such as osteosarcoma. Additionally, GLCM and First Order features like “HighGrayLevelEmphasis” reveal the importance of high gray-level values, possibly indicating harder tumor regions, typical in osteosarcoma, reflecting the presence of calcified or ossified areas within the tumor.

## 4 Discussion

In recent years, the application of Computer-Aided Diagnosis (CAD) technology in medical imaging has rapidly advanced, leading to continuous improvement in accuracy (16). More hospitals are introducing CAD-related applications, enhancing work efficiency, reducing the workload of physicians, and alleviating work intensity. Developing radiomic-assisted models for the diagnosis of osteosarcoma and chondrosarcoma, based on medical imaging, can alleviate the workload of radiologists. It enables timely and effective screening of tumor types in primary hospitals, providing patients with the most timely and accurate diagnostic opinions, thereby avoiding missing the optimal diagnosis timing or unnecessary treatment plans (17–20). In this study, we aimed to explore and validate the effectiveness of using machine learning models combined with radiomic features to accurately distinguish between osteosarcoma and chondrosarcoma. By extracting high-dimensional radiomic features from CT images, this study comprehensively trained and evaluated six advanced machine learning algorithms. The results showed that the RF model performed the best among all tested models, with an AUC value reaching a perfect 1.00, indicating extremely high classification accuracy and reliability. Furthermore, SHAP value analysis provided insights into the decisive role of specific radiomic features in these models, enhancing the interpretability and clinical application potential of the models.

In this study, the performance of the RF algorithm surpassed that of other machine learning models. Several factors contribute to this superiority. Firstly, RF is an ensemble learning technique that enhances prediction accuracy and stability by building multiple decision trees and averaging or majority voting their results. This approach is particularly effective in handling high-dimensional data, as it can reduce the risk of overfitting while maintaining sensitivity to hidden patterns in the data. For the task of distinguishing between osteosarcoma and chondrosarcoma, the high dimensionality and complexity of radiomic features demand algorithms that can effectively manage a large number of input variables and extract key information from them. RF ensures model robustness by introducing randomness in feature selection during the construction of each decision tree, preventing overreliance on certain features and enhancing the model’s generalization ability. Additionally, the RF algorithm internally assesses feature importance, enabling the model to automatically identify radiomic features most influential for classification. Moreover, the RF model demonstrates robustness in handling imbalanced datasets, a common challenge in clinical data where one class may outnumber the other. This robustness is critical for accurately classifying osteosarcoma and chondrosarcoma, given their clinical data often exhibit imbalances between the two types.

Based on SHAP analysis, we uncovered associations between radiomic features and imaging characteristics of osteosarcoma, enhancing clinical interpretability and summarizing the crucial role of radiomic features in discriminating between osteosarcoma and chondrosarcoma. We found that features such as GLCM and first-order statistical features from wavelet transformations are essential for identifying tumor microstructure and macroscopic characteristics. These features reflect the complex texture and density distribution within the tumor, with “DependenceLowGrayLevelEmphasis” revealing the pixel dependency of low gray-level regions, possibly associated with necrotic areas of the tumor, and “JointAverage” providing information about the overall grayscale level of the tumor, aiding in assessing overall density and calcification. Such information is challenging to derive from traditional imaging techniques, demonstrating the value of radiomics in modern healthcare. Through this technology, physicians can better understand the malignancy and biological characteristics of tumors, making more accurate clinical decisions and treatment plans.

This study, by combining advanced radiomic features and machine learning techniques, enhances the diagnostic accuracy of osteosarcoma and chondrosarcoma, which is significant in clinical practice. Particularly for tumors like osteosarcoma, with high heterogeneity and complex local manifestations, traditional imaging methods provide useful information but have limitations, especially in detecting early and subtle lesions (21). By leveraging machine learning techniques to analyze radiomic data, this study reveals subtle texture and morphological changes critical for distinguishing tumor types, which may not be evident to the naked eye. Early diagnosis and accurate tumor typing are crucial for improving treatment planning and prognosis for osteosarcoma patients. Accurate imaging analysis can help physicians decide whether to perform a biopsy, select the type of surgery, and determine the need for adjuvant chemotherapy or radiotherapy. For instance, accurate identification of highly malignant osteosarcoma through radiomic features allows patients to receive more aggressive treatment early, thereby improving survival rates (22).

Compared with existing studies, this study has certain characteristics in methods and applications (23). For example, Zheng et al. (15) used fusion imaging omics features to predict the response to neoadjuvant chemotherapy for osteosarcoma, while Luo et al. (24) predicted synchronous lung metastasis of osteosarcoma through multi-parameter MRI imaging omics analysis. These studies mainly focus on the prediction of specific treatment responses or metastasis risks, while this study focuses on the type distinction between osteosarcoma and chondrosarcoma, providing an innovative imaging omics method for the preliminary classification of osteosarcoma malignant tumors. The highlight of this study is that multiple machine learning models were used for comparison, and the interpretability of the model was enhanced through SHAP analysis, thereby ensuring that the model has high performance and interpretability for clinical application. In addition, the data of this study came from two independent centers. Although the sample size was limited, the robustness of the model was improved through rigorous cross-validation and feature selection.

Although this study has achieved some success, it also has limitations. Firstly, the study relies on retrospective CT data from two centers, which may limit the generalizability and extrapolation of the findings. The total number of patients included in this study was also limited, which may affect the generalizability of the model results. Future research needs to validate these findings on a broader dataset to ensure the stability and reliability of the models across different populations and devices. Secondly, this study primarily focuses on the performance of machine learning models and pays less attention to other clinical features of patients, which may affect tumor imaging characteristics and treatment response. Incorporating these clinical variables into the model may further improve diagnostic accuracy and relevance. Additionally, while SHAP values provide interpretability of model decisions, explaining how these radiomic features correlate with specific biological characteristics of tumors remains a challenge. Further biomedical research is needed to explore the exact relationship between these radiomic markers and tumor behavior. Future work should focus on expanding the sample size of the study, incorporating more clinical variables, and optimizing the practicality and efficiency of the algorithms, so that these advanced machine learning techniques can better predict osteosarcoma and chondrosarcoma.

## 5 Conclusion

This study aimed to explore the use of machine learning combined with radiomic features to improve the differentiation accuracy of osteosarcoma and chondrosarcoma. By extracting high-dimensional radiomic features from CT images and applying six machine learning models for training and evaluation, the results showed that the RF model performed the best among all tested models, while other models also demonstrated high diagnostic accuracy. Additionally, SHAP value analysis enhanced the interpretability, providing clinicians with the most critical radiomic features for tumor type diagnosis. Overall, this study confirms the effectiveness of combining machine learning and radiomic features in the classification of osteosarcoma and chondrosarcoma, demonstrating the potential of this approach in improving diagnostic accuracy and interpretability.

## Data Availability

The data analyzed in this study is subject to the following licenses/restrictions: The datasets used and/or analyzed during the current study are available from the corresponding author upon reasonable request. Requests to access these datasets should be directed to Xiao-Bin Tian. E-mail: ltxb6@vip.163.com.
